# Operationalizing the principles of patient engagement through a Patient Advisory Council: Lessons and recommendations

**DOI:** 10.1111/hex.13909

**Published:** 2023-11-09

**Authors:** Ingrid Nielssen, Maria Santana, Surakshya Pokharel, Kimberly Strain, Veronika Kiryanova, Sandra Zelinsky, Zoha Khawaja, Prachi Khanna, Anni Rychtera, Anshula Ambasta

**Affiliations:** ^1^ Strategy for Patient Oriented Research (SPOR) Support Unit Edmonton Alberta Canada; ^2^ Department of Medicine, Cumming School of Medicine University of Calgary Calgary Canada; ^3^ Ward of the 21st Century, Calgary Zone of Alberta Health Services University of Calgary Calgary Canada; ^4^ Department of Biology, Faculty of Science University of British Columbia Vancouver Canada; ^5^ Strategy for Patient Oriented Research (SPOR) Support Unit Vancouver British Columbia Canada; ^6^ Department of Anesthesia, Pharmacology and Therapeutics, Therapeutics Initiative University of British Columbia Vancouver Canada; ^7^ Department of Medicine, Cumming School of Medicine University of Calgary Calgary Alberta Canada

**Keywords:** co‐design, patient engagement, Patient Engagement Framework, patient research partner

## Abstract

**Background:**

Inclusiveness, Support, Mutual Respect and Co‐Build are the four pillars of patient engagement according to the Strategy for Patient‐Oriented Research (SPOR). The aim of this manuscript is to describe the operationalization of these principles through the creation of a Patient Advisory Council (PAC) for the research study titled ‘Re‐Purposing the Ordering of Routine laboratory Tests (RePORT)’.

**Methods:**

Researchers collaborated with the Alberta SPOR SUPPORT Unit (AbSPORU) Patient Engagement Team to create a diverse PAC. Recruitment was intentional and included multiple perspectives and experiences. PAC meetings were held monthly, and patient research partners received support to function as co‐chairs of the PAC. Patient research partners were offered training, support and tailored modalities of compensation to actively engage with the PAC. Regular member check‐ins occurred through reflexivity and a formal evaluation of PAC member engagement.

**Results:**

The PAC included between 9 and 11 patient research partners, principal investigator, research study coordinator, improvement scientist, resident physician and support members from the AbSPORU team. Twelve monthly PAC meetings were held during the first phase of the project. The PAC made course‐changing contributions to study design including study objectives, recruitment poster, interview guide and development of codes for thematic analysis. Patient research partners largely felt that their opinions were valued. Diversity in the PAC membership enhanced access to diverse patient participants. Furthermore, support for co‐chairs and patient research partner members enabled active engagement in research. In addition, a culture of mutual respect facilitated patient partner engagement, and co‐design approaches yielded rich research outputs.

**Conclusions:**

Collaboration between research teams and Patient Engagement Teams can promote effective patient engagement through a PAC. Deliberate and flexible strategies are needed to manage the PAC to create an ecology of Inclusiveness, Support, Mutual Respect, and Co‐Build for meaningful patient engagement.

**Patient or Public Contribution:**

Patient research partners were involved in the decision to write this manuscript and collaborated equitably in the conception and development of this manuscript, including providing critical feedback. Patient research partners were active members of the PAC and informed the research project design, participant recruitment strategies, data collection and analysis, and will be involved in the implementation and dissemination of results. They are currently involved in the co‐development of a patient engagement strategy using a Human‐Centered Design process.

## 1 INTRODUCTION

Patient engagement in health research is an established response to the imperative that those being researched have a voice in the research that impacts them.^1^, ^2^ In 2011, the Canadian Institutes of Health Research (CIHR) introduced the Strategy for Patient‐Oriented Research (SPOR) initiative, with the aim of bringing patient perspective into health research.^3^ CIHR defines patient engagement as activities that happen when patients, family members, caregivers, friends and specific affected communities (collectively ‘patients’) collaborate as active and equal research partners on health research projects.^3^ Patient engagement is authentic when patients and communities have the capacity and support to be equitably engaged in research; and when researchers recognize the value of patient inclusion in research and have the capacity to effectively collaborate with patient research partners.^4^, ^5^ Including patients as partners in research ensures that research findings reflect patient priorities, that research processes and activities are more accessible to often missed communities and populations, and that research results can be implemented sooner and in more useful ways.^1^, ^5^, ^6^, ^7^ Patient engagement can have positive impacts on patient partners, research processes, and research outputs.^8^ A scoping review that evaluated the impact of patient engagement on healthcare quality indicated that involving patients in improvement projects can increase the quality of care and help identify new ways of providing care.^9^ For example, involving patients in self‐monitoring, or documenting blood pressure reading was associated with an increased likelihood of reaching the target blood pressure.^10^ In another example, adding a patient engagement tool to positive airway pressure treatments may help adherence to treatment and reduce mask leakage.^11^ Patients need to be engaged early, often, and at as many stages of the research cycle as possible.^3^

Foundational to the CIHR SPOR Patient Engagement Framework are the four principles of Inclusiveness, Support, Mutual Respect and Co‐Build.^12^ However, literature on how these principles may be applied pragmatically is scarce.^13^, ^14^ Understanding how each of these principles may be operationalized within the practical limitations and timelines of funded research projects can contribute to the advancement of patient engagement in health research. The establishment of Patient Advisory Councils (PACs) is a strategy to integrate patient engagement into research. Establishing and actioning PACs within the CIHR SPOR Patient Engagement Framework can meet the relational and reciprocal needs of research teams and contribute to a collaborative and patient‐centred research ecology. PACs can provide flexible spaces for patient research partner engagement, offering opportunities for training, support and collaboration throughout the research project continuum. Though there exist guidelines and recommendations on ‘good patient engagement practices’^5^, ^7^ on researcher and patient research partner competencies, and on the creation of inclusive and welcoming collaborative spaces,^15^, ^16^, ^17^, ^18^ evidence on how PACs specifically can be established and sustained through the application of patient engagement principles is scarce. There is little known about the role and outputs of PACs in relation to the research team, and how PACs can effectively advance the science and practice of patient engagement in health research.^13^, ^14^, ^19^ A 2018 systematic review on PACs concluded that there is a paucity of evidence to guide strategies for engagement through PACs and a need for standardized reporting of recruitment and engagement strategies.^20^

CIHR has established SPOR ‘*S*upport for *P*eople and *P*atient‐*O*riented *R*esearch and Trials’ (SUPPORT) Units across Canada, which provide support to research teams to advance the science and practice of patient engagement.^21^ In Alberta, the SPOR SUPPORT Unit (AbSPORU)^22^ has a Patient Engagement Team which established a PAC to support a research programme titled ‘Re‐Purposing the Ordering of Routine laboratory Tests’ (RePORT) in hospitalized medical patients. This research programme aims to reduce the overuse of daily repetitive laboratory testing in hospitalized medical patients using a deimplementation strategy that will combine healthcare provider engagement tools with patient–partner‐designed patient engagement tools. In this manuscript, we describe how we actioned the principles of the SPOR Patient Engagement Framework to establish a PAC, present our experiences and lessons learned, and provide concrete suggestions for teams looking to build PACs as inclusive and supportive spaces for patient‐oriented co‐build work.

## 2 METHODS

### 2.1 Setting

The academic research team collaborated with AbSPORU to establish a PAC that would support the RePORT research programme in progressive phases. In Phase 1, the patient research partners worked with the research team to systematically understand the patient and family/caregiver's participant experience and perspectives on routine blood testing in hospitals and supported the research team in seeking grant funding for further aspects of the project. During Phase 2, patient research partners are using information obtained from Phase I to co‐develop a patient‐engagement strategy that can help patients be more informed of the laboratory blood testing process. In the future, the patient research partners will support the research team with the implementation of an intervention bundle (including the co‐developed patient engagement strategy) across multiple hospitals. Patient research partners will also support the research team with evaluation of the impact of the intervention bundle, and dissemination of results. In this manuscript, we focus on Phase 1 of the project which ran from May 2021 to June 2022.

### 2.2 PAC composition and structure

To recruit patient research partners, we disseminated a recruitment letter widely through online patient and community networks such as Albertans4HealthResearch.ca,^23^ Patient Voices Network in British Columbia^24^ and Alberta and British Columbia SPOR SUPPORT Units. We also used snowballing through networks of existing patient research partner PAC members to continue to build the PAC. Our PAC included 9–11 patient research partners who represented diversity in lived experiences of multiple health conditions, healthcare research experience, and reflected diverse academic and professional backgrounds (Table 1). These patient research partners included three graduates, and two current students, from the Patient and Community Engagement Research programme, an experiential learning programme offered through the University of Calgary Continuing Education^25^ that teaches participatory health research methodologies to patients and community members. The AbSPORU Patient Engagement Team provided support through three additional team members, including a patient research partner lead who was also a co‐investigator on the initial grant application, a patient engagement coordinator, and a research and evaluation coordinator. The PAC also included the research team's Principal Investigator, a Research Study Coordinator, an expert in Improvement Science and a resident physician in training. As the project developed, we recruited a Human‐Centered Design specialist to our team and a Patient Engagement summer student to support the evaluation of patient engagement. Two patient research partners who were nominated by the study Principal Investigator and the AbSPORU Patient Engagement Team, along with the research study coordinator, served as the co‐chairs of the PAC.

**Table 1:** Patient Advisory Council (PAC) composition and structure.

Demographic characteristics	Number within the PAC
PAC role	
Patient research partner	8
Academic research partner	2
Age, years	
18–29	3
30–49	2
60–64	4
65+	1
Gender	
Man	2
Woman	8
Race	
East Asian	1
South Asian	2
Southeast Asian	1
White	6
English as a first language	
Yes	6
No	4
Living with a chronic health condition?	
Yes	9
No	1
Research experience	
New to research	1
Moderate research experience	5
Extensive research experience	3
Prefer to not say	1

Based on a total of 10 survey responses from all PAC members which includes eight patient research partners.

### 2.3 PAC function

**Figure 1:**
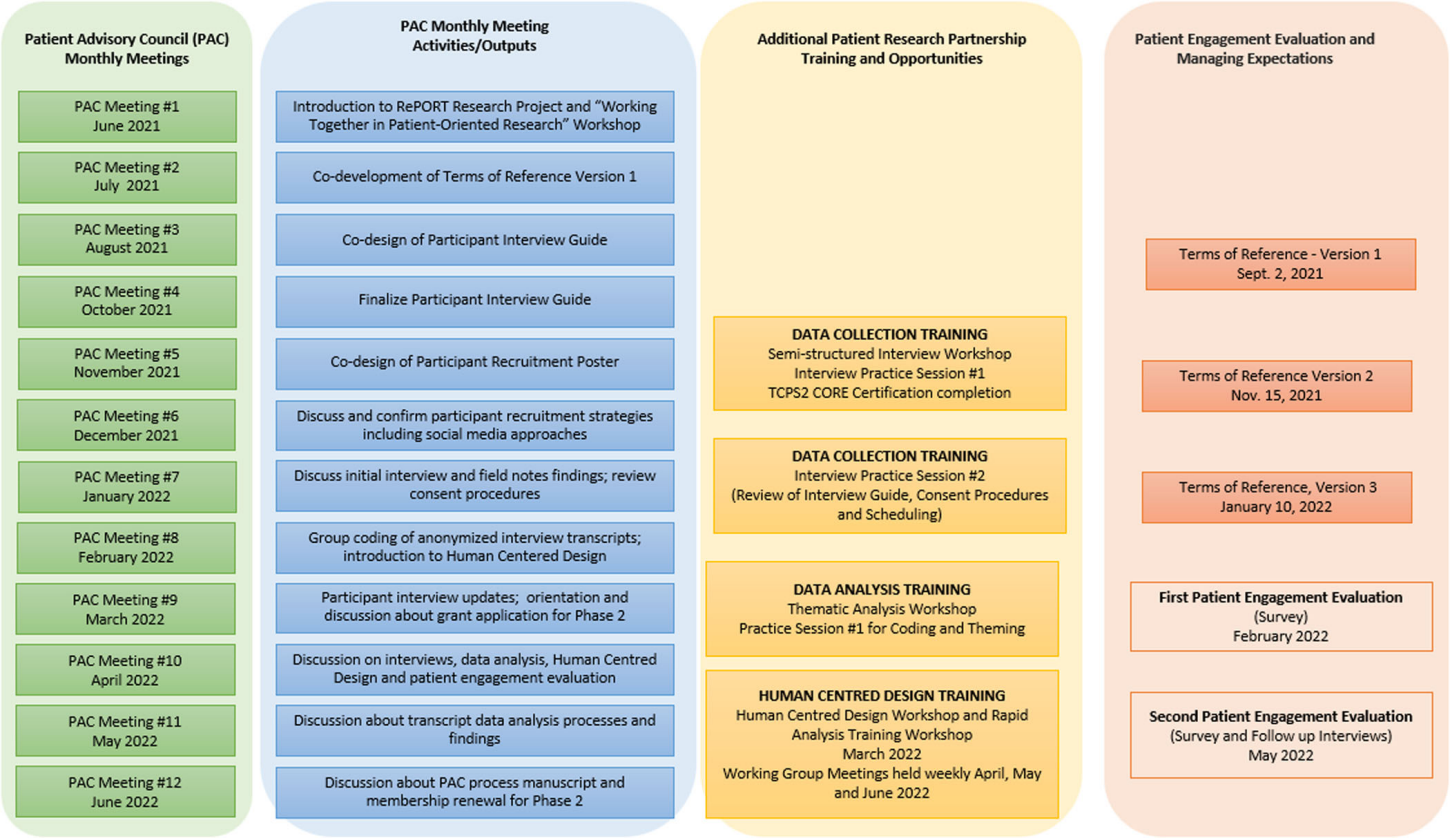
A diagram of the different outputs of the Patient Advisory Council (PAC) along with patient research partner opportunities during the research study period.

We conducted 12 monthly PAC meetings in Phase 1 of the project (Figure 1) and held them online via a virtual meeting platform—Zoom Video Communications Inc.^26^ We recorded the meetings with the permission of the PAC members. The monthly PAC meetings were scheduled for 90 min with a mid‐way 5‐min break. The meetings began with a welcome, introductions of any new members and an icebreaker. The ice‐breaker section of the monthly meetings usually ran for at least 10 min. The ice‐breaker topics (e.g., please identify one object in your freezer that is unique) were meant to connect and share the unique perspectives and identities each member brought to the team. In addition, in each meeting, we included updates from the Principal Investigator and the AbSPORU Patient Engagement Team about project status, aspects of patient research partner and participant recruitment, evaluation of patient engagement and patient research partner training and compensation. We established a shared online folder on Google Drive^27^ to house meeting agendas, notes and recordings of meetings, membership lists, updated versions of Terms of Reference, members' biographical pages and other project‐related documents. To integrate patient perspective into the project work and outputs, three working groups were formed—Data Collection Working Group, the Data Analysis Working Group and the Human‐Centered Design Working Group.

The first PAC meeting included a ‘Working Together’ workshop delivered by the patient research partner lead and research and evaluations coordinator. The goals of the workshop were to describe patient‐oriented research in the context of the CIHR Framework, describe what ‘lived experience’ as expertise meant and explain the roles of patients as partners in research. The second PAC meeting included the co‐development of the Terms of Reference for the PAC. In addition to regular updates and opportunities for questions and discussion, each subsequent PAC meeting included a collaborative group activity relating to project processes. These included the co‐development of a participant recruitment poster, co‐design of the participant interview guide and working to identify codes on transcripts that subsequently informed codebook development (Figure 1). Members were kept engaged using Zoom Breakout Rooms^26^ and Jamboard,^28^ and every effort was made to ensure PAC members were comfortable with these new online approaches. Zoom Breakout Rooms is a feature on Zoom Video Communications Inc.,^26^ which allows participants to be divided into smaller subgroups to foster greater engagement and discussion. Jamboard^28^ is an interactive, collaborative, digital whiteboard developed by Google that makes sharing ideas in real‐time easier.

The ‘Working Together’ workshop was followed up with multiple optional research skill‐building workshops. The academic researchers in collaboration with AbSPORU Patient Engagement Team organized two 90‐min training workshops on qualitative methods for data collection from semistructured interviews. One session discussed how to support participant interviews, and the other session included practice interview sessions. The co‐developed interview guide was shared before these sessions. The practice interview sessions were held virtually where members of the workshop practiced being both facilitators and interview participants. We also organized two training workshops on qualitative data analysis. The first session was 2.5 h long, which discussed how qualitative analysis is conducted, and included a practice coding session. Members were provided with a short excerpt from one of the interviews so that they could practice coding in smaller groups first, and then reconvene to collectively discuss with the rest. Members were also given ‘homework’ to practice coding and we held a subsequent 60‐min session to discuss the coding from the homework. In addition, a workshop was conducted to introduce patient research partners to the concepts of Human‐Centered Design to build capacity for the development of patient engagement tools in Phase 2 of the project. While participation in the workshops was optional, completion of the workshops was necessary to engage in the respective aspects of the project.

All patient research partners who conducted data collection and analysis completed the Tri‐Council Policy Statement Course on Research Ethics 2 Certificate^29^—a requirement in Canada for collecting data from human research participants. This is an online self‐paced course featuring interactive exercises and multidisciplinary examples that help familiarize researchers with ethical principles pertaining to research.

We had 16 members in the Data Collection Working Group, 11 members in the Data Analysis Working Group and 13 members in the Human‐Centered Design Working Group. All members participated actively during the interactive sessions including small group brainstorming sessions, Google Jamboard use,^28^ Zoom breakout rooms^26^ and so forth. Patient research partner attendance at each of the research skill‐building workshops was more than 70% of our membership. At each PAC meeting, at least 50% of PAC members were in attendance.

In Phase 1, an evaluation of patient engagement was conducted, using two surveys (initial and follow‐up) based on the Patient and Public Engagement Evaluation Tool,^30^ followed by semistructured interviews conducted by a summer student external to the PAC (Figure 1).

### 2.4 Patient research partner compensation

Patient research partners were offered compensation for their time preparing for and attending meetings and workshops, responding to surveys and evaluations, reviewing any project‐related documents and engaging in project processes such as data collection and analysis. The rate for compensation was guided by the ‘AbSPORU Patient Partner Appreciation Guidelines’ (Appendix Item S1). Confidential conversations regarding compensation took place between AbSPORU Patient Engagement Team member and each patient research partner individually, at the beginning of the project and at scheduled intervals. Compensation discussions would include patient research partners' unique circumstances, and preferences regarding payment. Individualized ways of payments were made in formats that supported the patient research partner's income reporting requirements and also aligned with institutional payment processes. A Compensation Submission Form (Appendix Item S2) was created for patient research partners to submit their hours during each payment term.

## 3 RESULTS

### 3.1 PAC contributions towards patient engagement

The PAC worked together with the research team to co‐build a Terms of Reference Document (Appendix Item S3) to establish and manage member expectations, and to guide the work and role of the PAC in relation to the research programme. Synergistic to this work, the AbSPORU Patient Engagement Team was able to update existing relevant patient‐oriented research resources including templates for PAC meeting Agendas/Notes and Action Items, Terms of reference template, AbSPORU patient research partner appreciation guidelines, patient engagement cost calculator to support patient engagement budget on grant funding applications, patient research partner institutional payment guidelines, and a patient research partner compensation tracking and submission form.

### 3.2 PAC contributions to the RePORT research programme

With respect to supporting Phase 1 of the project, PAC members worked with the research team members to obtain ethics approval for a study to qualitatively understand patient and family/caregiver participants' needs and perspectives with respect to inpatient laboratory testing. PAC members helped in the design of the study protocol, informed consent form, participant recruitment poster, and interview guide (see Figure 1). Specifically, the patient research partners suggested changing the study objective from understanding barriers and facilitators around appropriate laboratory testing to understanding the current experience and perspectives of patients and families/caregivers. They also were instrumental in designing a recruitment poster that used patient‐centred language and co‐developing the interview guide through several cycles of revisions.

Patient research partners were also involved as co‐interviewers and in the development of the codebook. Six unique patient research partners collaborated as co‐interviewers in 12 interviews. Six patient research partners participated in the development of the codebook for thematic content analysis. The process of developing the codebook took place over several steps. The research study coordinator coded an initial transcript with one patient partner. The Data Analysis Working Group including patient research partners and the research study coordinator, then coded two full transcripts. The codes identified from this work (that included codes from six patient research partners' coding) were then used to create the codebook. To facilitate easier participation of patient research partners, no software was used for this step of analysis.

PAC members also joined the research team on a grant funding application to obtain funding for subsequent planned phases of the project. The AbSPORU Patient Engagement Team provided support for this, including help with obtaining research logins and accounts and completion of letters of support. The co‐authored grant funding application was successful, with the involvement of patient research partners being considered a key strength of the application. One challenge faced during the application process was being able to enroll all PAC patient research partners as co‐investigators on the application. Owing to time constraints and requirements of unique research logins and letters of support, a smaller number of interested patient research partners including PAC co‐chairs were included in the application.

Currently in Phase 2 of the project, nine patient research partners are engaged in working with a Human‐Centered Design specialist in a specific working group to develop a patient‐engagement strategy around understanding appropriate laboratory blood testing processes for patients and families/caregivers in hospitals. This group is using a human‐centred process, while leveraging design thinking methodologies to discover themes from rapid analysis of interview transcripts, define existing knowledge needs, and design intervention tools to fulfil these needs. During rapid analysis, patient research partners reviewed audio and written transcripts of interviews and pulled out key insights from direct quotes. These insights outlined a specific need and were then categorized into themes. Keeping these themes in mind, this group has co‐designed an infographic, a video and a website meant to engage patients with blood testing in hospitals. Patient research partners actively conceived of and designed these tools, with a keen eye on appropriate wording and sensitive images (e.g., excluding an image of a sharp needle). They are currently working on evaluating these tools and designing an implementation strategy for these tools.

### 3.3 PAC engagement evaluation

Nine patient research partners participated in the baseline survey and six participated in the follow‐up surveys. Nine patient research partners subsequently participated in semistructured interviews conducted by an AbSPORU summer student external to the PAC. A detailed evaluation of engagement is published separately.^31^ Overall, patient research partners agreed that the support (e.g., training and compensation) needed to contribute to the project was available. Most patient research partners felt that their opinions and views were heard, however they provided some suggestions for improving diversity and collaboration within the various PAC working groups.

### 3.4 PAC findings

Findings from the process of establishing this PAC are summarized in Table 2, and below, according to the guiding principles of Inclusiveness, Support, Mutual Respect and Co‐build from the CIHR Patient Engagement Framework.^12^

**Table 2:** List of recommendations to operationalize each fundamental principle of the Canadian Institute of Health Research's Patient Engagement Framework in a Patient Advisory Council (PAC).

Fundamental patient engagement principles	Study findings	Recommendations for PACs
Inclusiveness	Diversity in PAC membership enhanced reach and access to diverse patient participants, increasing the generalizability of research findings	1. Broad and targeted outreach to invite members from multiple communities and backgrounds, as well as various lived experience of health conditions and partnered research. 2. Recruitment of enough members to account for possible future absenteeism and attrition owing to unique health and other circumstances of diverse members. 3. An open membership policy that is responsive to members’ and research programme's needs. 4. Regular evaluation to ensure PAC members their engagement is meaningful. 5. Consistent and comprehensive communication strategies and clear description of roles 6. Meeting times that are flexible and accommodate the physical and geographical circumstances of members. Additional ways to support missed meetings include an online shared documents folder, meeting recordings, etc. 7. Concerted efforts and dedicated meeting time for building relationships of trust that are essential to meaningful and effective research outputs (e.g., icebreakers, biography page, in‐person events when possible).
Support	PAC co‐chairs and members benefitted from direct support with specific tasks including agenda‐building and note‐taking; dedicated research skill‐building workshops, and compensation for their time. The enhanced support led to enhanced involvement in the research programme	1. Adequate budget and timelines to support the resources and time required for patient research partner collaboration and training. Include patient engagement budget in grant funding applications. 2. Collaboration with dedicated patient‐oriented research teams (e.g., Strategy for Patient‐Oriented Research Support Units) to support patient research partner engagement and collaboration. 3. Dedicated support to enable success of patient research partner co‐chairs and members including sharing of established templates for agenda‐setting and note‐taking, Terms of Reference and patient research partner compensation processes. 4. Direct peer‐to‐peer support between patient‐research partners and research personnel for understanding research processes, relationships and landscapes. 5. Specific research process training workshops for all patient research partners (e.g., workshops on conducting interviews and thematic data analysis) without patient research partner obligation to commit to conducting these aspects of research projects. 6. Personalized, flexible and adaptable compensation processes for patient research partner contribution and collaboration on research project design, processes and activities.
Mutual respect	Establishing a culture of mutual respect facilitated patient partner engagement with PAC activities and working groups	1. Clear communication about role, expectations, time commitment, training and compensation being offered for patient research partner per activity. Encouragement of peer mentorship between patient research partners. 2. Transparency in communication about project progress and any changes (e.g., regular updates and sharing of project documents and information) can assure meaningful engagement and trust. 3. Support for early discussions about working together and co‐building Terms of Reference to explain project background, scope and timelines, to establish meeting guidelines and to confirm member expectations, roles and responsibilities. 4. An introductory workshop to establish and share the principles of patient‐oriented research including the core principles of working together in respectful and mutually beneficial ways. 5. Identification and provision of training and capacity‐building workshops to patient research partners.
Co‐build	Co‐design approaches yield rich research outputs with more patient‐centred language and relevant tailored details	1. Early inclusion of patient research partners in co‐building project processes and documents such as a living Terms of Reference document. 2. Graduated support with co‐building. Socialize and establish democratic co‐building processes. Recognizing interest, motivations and opportunities may change as the project progresses. 3. Building capacity in the use of tools (e.g., JamBoard, Google documents) to support multiple people working together on a document and at times suitable to them. 4. Use of group time during meetings to allow for relationship building and generative discussion. ‘Working together’ on documents and activities can socialize awareness of multiple perspectives and assure they are reflected in research outputs. 5. Clear communication and regular updates on project timelines and deadlines can assure all member are regularly included in project activities and projects are completed in a timely matter. Commit to regular feedback and follow‐up loops.

#### 3.4.1 Inclusiveness

Inclusiveness is about bringing together multiple perspectives and creating a supportive environment that recognizes and respects unique ways of living, being and learning. To operationalize this principle, several strategies were used. For instance, members from diverse backgrounds were recruited through a broad and targeted outreach, while ensuring enough members were recruited to account for possible future absenteeism or attrition. In addition, an open membership policy was issued, consistent and comprehensive communication strategies were used (e.g., online accessible folders for nonsensitive information), role descriptions were developed (through Terms of Reference documents), flexible meeting times were set, and a portion of meeting time was dedicated for building relationships (e.g., icebreakers, interactive online sessions, members sharing biography pages, etc.). PAC members identified with various experiences of health issues and challenges and represented multiplicity with respect to different academic and professional backgrounds. This diversity enhanced our reach in accessing research participants from various communities. The peer‐to‐peer interviews and patient research partner collaboration on data analysis ensured our results were more universal.

#### 3.4.2 Support

To ensure adequate support for patient research partners, our initial project budget and timelines included the patient engagement component. The AbSPORU Patient Engagement Team members worked closely with the research team to provide support to the patient research partner co‐chairs, including the development of templates for compensation processes, agenda‐building and note‐taking. This support gradually decreased with increasing independence of the patient research partner co‐chairs. For example, for the first several months, additional co‐chair meetings were held on a weekly basis, with a gradually decreasing frequency based on the decreasing need for support from our patient research partner co‐chairs. The patient research partner lead discussed compensation with each patient research partner individually at the beginning of their engagement with the project, aligned with guidelines in the compensation conversation.^32^ Patient research partners tracked their own hours and submitted a standardized payment submission form that also included a preferred method of payment. Methods of payment included honoraria, contract, casual hire and pre‐paid gift cards. The AbSPORU Patient Engagement Team also provided multiple research skill‐building workshops to enable patient research partners to collaborate on co‐conducting interviews, data analysis and Human‐Centered Design of the patient engagement strategy. Guidance about expectations and hours that could be claimed for additional activities outside of PAC meetings (such as meeting or workshop preparation time, additional time spent on data collection, data analysis and co‐design of research tools) was provided by the research team.

As a result of these efforts to provide support to the patient research partners, we had excellent collaboration with them in all our research processes including study design, development of interview guides and recruitment posters, participation in interviews and engagement in thematic and rapid analysis of transcripts.

#### 3.4.3 Mutual respect

Mutual respect is about valuing unique and individual expertise and experiential knowledge.^12^ Intentional strategies to create a culture of mutual respect were used. After the introductory ‘Working Together’ workshop introduced the fundamental pillars, one of the first co‐build projects worked on by the PAC was the development of a Terms of Reference document. This document contained a clear description of roles, training offered, expected time commitments and compensation being offered. A shared accessible online folder was established to ensure accessibility and transparency about project progress and relevant changes. Early discussions with patient research partners were held to understand what kinds of support responded to their voiced needs. Opportunities for peer mentoring were also offered including where one patient research partner was able to support another in building skills in a specific area. This environment of mutual respect facilitated patient engagement as noted through participation in the monthly PAC meetings, as well as the skill‐building workshops.

#### 3.4.4 Co‐build

To develop skills and processes for co‐building, patient research partners were included early on with co‐building of the Terms of Reference document. Capacity was built with the use of tools such as JamBoard and Google documents that would support multiple people asynchronously working together, the use of group meeting times to allow for generative discussion and clearly communicated deadlines for completion of documents. Graduated support was provided with co‐building work, and democratic and respectful co‐building processes were established. The co‐build outputs of this PAC included the Terms of Reference document, participant recruitment poster, interview guide, a codebook for thematic analysis, co‐partnership in a project grant application and co‐authorship of this publication. The co‐designed materials benefitted with more patient‐centred language, appropriate visuals and details that were more likely to elicit insights from patient experience. Patient research partners contributed to making our results more generalizable, and with the dissemination of those results, including in their capacity as manuscript co‐authors.^33^

## 4 DISCUSSION

The establishment and functioning of this PAC embody the operationalization of the principles of the CIHR SPOR Patient Engagement Framework. The described PAC model offers an innovative approach to effective and sustained engagement with patients as partners throughout the continuum of a patient‐oriented research project. This model provides evidence of the meaningful contribution SPOR SUPPORT Units can provide in improving patient engagement in health research. In the literature, PACs are described as facilitators for patient engagement, and a way to enhance patient voice and input to co‐produce patient‐centred research that can ultimately affect policy priority and health system change.^6^, ^8^, ^13^, ^14^, ^19^ While other studies have used similar PACs in Canada,^13^, ^34^ our PAC model is unique in the degree of patient research partner engagement (e.g., co‐chairing meeting and developing own agendas, leading meetings, co‐building documents and research outputs), the extent of supports provided for research (e.g., co‐conducting interviews, participating in thematic and rapid analysis of transcripts), and conduct of a formal patient engagement evaluation.

Literature has previously identified that being flexible with meeting times and providing support for medical and personal conditions are essential factors for promoting inclusive patient engagement.^6^ Prior studies show that merging scientific knowledge and research expertise with unique patient research partner lived experience can help improve the validity, rigour and credibility of research.^8^ This can be further enhanced if patient research partners bring multiple social, cultural and economic perspectives, as well as differing lived experiences and insights about the health conditions and experiences that impact them. Our findings are thus consistent with what has previously been described in the literature. Existing literature also shows that support and training are elemental facets of working together meaningfully and effectively in co‐production work.^35^, ^36^ Resources and adequate time for relationship building have been identified as important elements in co‐production projects.^17^ Lack of funding can make it difficult to offer sufficient resources to support patient research partner training in research. In the absence of training, patient research partners feel overburdened and feel unable to contribute adequately.^37^ Facilitators for patient engagement in our project were time and the availability of funding from CIHR. Compensation is essential to recognizing patient research partner contributions to health research projects.^38^ Similar to our findings, patient research partner relationships are known to flourish in a culture of trust.^39^ Transparency is essential to trust and considered relational and social processes are essential in fostering effective engagement within research teams.^16^, ^18^ Consistent with our study findings, co‐design approaches are known to yield better research results and outputs with more universal applicability.^40^

Our study further advances the literature in this area by providing concrete and pragmatic approaches that can be used to build inclusive and welcoming PACs (Table 2). In addition, a formal evaluation of patient engagement within this PAC has been published.^31^ Future areas of research to further the science of patient engagement will be in sustaining motivation and partnership from patients over long‐term research projects, development of standardized measures of patient involvement in research, and evaluation of the specific impact of patient research partners' research activities. The findings from this study indicate that healthcare organizations, research institutions and policymakers seeking to improve patient engagement in health research need to advocate for several strategies to ensure the sustainability of research with patient research partners such as: research funding that incorporates a patient engagement budget and timelines compatible with true co‐build work, the development of dedicated Patient Engagement Teams (e.g., SPOR SUPPORT Units) to support research teams, as well as the promotion of platforms for co‐dissemination of results. In the long term, the PAC model provides the necessary infrastructure, appropriate support and access required for patient research partners to engage in meaningful research with thriving research outcomes.

### 4.1 Challenges and next steps

Patient research partners come to projects with differing motivations and expectations which can evolve over time. Moreover, the health conditions that draw patient research partners to projects can also pose challenges to their sustained engagement. This specifically contributed to the attrition of members of our council. Members of this PAC represented a broad range of health conditions and health research experience but did also represented people with the privilege of having the technological access required for engagement. Their experience with the health care system translated to exceptional familiarity with hospitalization and blood testing procedures. In the future, it would be valuable to include additional members who represent greater diversity in health literacy and access. It was not always possible to maintain a balance between project timelines, budget and patient research partner availability. It takes intentional work and planning from research teams to ensure opportunity and support for patient research partner engagement at all phases of designing and conducting participatory health research projects. For instance, patient research partners collaborated in codebook creation and rapid analysis but owing to the timeline and budgetary constraints, the research team was not able to support a more in‐depth engagement of patient research partners in thematic analysis.

There was significant work involved in navigating institutional systems to streamline compensation processes that were appropriate to each patient research partner's unique circumstance and preference. An outcome of this, however, was the establishment of a Patient Research Partner Compensation Process document specific to our institution that we have shared with other research teams that can have broad applicability to other institutions. The project did extend past its timeline and required an extension from the funding agency to accommodate the new timelines. Although accommodation of members' request for a more accessible folder for most project documents was done, sensitive documents (e.g., interview transcripts) were still on secure drives and accessibility remained an issue for those without institutional access to secure shared drives. The current processes of this PAC were developed by deliberate and systematic co‐learning with patient research partners; and listening to, identifying, responding to concerns and challenges and titrating resources based on their needs. To understand how to engage patient research partners effectively in the research process, the impact of their engagement on the patient research partners themselves must be taken into consideration.^5^

This PAC has currently developed patient engagement tools to support the RePORT research programme and is leading a formal evaluation of these tools. Some members are now part of a new Implementation Evaluation Working Group that is developing ways of implementing these tools and subsequently evaluating the implementation and effect of these tools. Two patient research partners are being supported to present this work at an international conference (Preventing Overdiagnosis Conference 2023) in Copenhagen. As patient research partners continue working through the different phases, more funds towards research skill development have been allocated for individual patient research partners. We also plan on re‐evaluating PAC member engagement at strategic phases over the research programme.

## 5 CONCLUSION

Meaningful collaboration of patient research partners and researchers in project processes and co‐building of outputs is best achieved and sustained through relationships fostered in ecologies of support, inclusivity, mutual respect, and flexibility. Guided by the principles of the CIHR SPOR Patient Engagement Framework, the innovative and unique approaches used by our PAC offer the possibility for flexibility, authentic inclusion and sustainability of patient research partner engagement across the continuum of health research projects. PACs can provide safe co‐learning spaces for patient research partners to receive training and support that enable them to share their lived experiences and provide additional expertise to research projects that matter to them. This leads to patient‐centred research processes that generate more patient‐centered results to improve healthcare access, practice, and policy for all.

### 5.1 Implications for researchers, practitioners and policymakers

The PAC model offers an innovative model for effective and sustained engagement of patients as partners in a patient‐oriented research project. We provide evidence of the contribution of dedicated patient engagement support units in improving patient engagement in health research. The patient research partners on our team received training and capacity building in research, which positions them well to potentially provide mentorship to patient‐oriented research teams more broadly going forward.

## AUTHOR CONTRIBUTIONS

Ingrid Nielssen, Maria Santana, Surakshya Pokharel and Anshula Ambasta have substantial contributions to the conception and design of the work and interpretation of data for the work. Kimberly Strain, Veronika Kiryanova, Sandra Zelinsky, Zoha Khawaja, Prachi Khanna and Anni Rychtera have substantial contributions to the conception and design of the work. In addition to the above, all authors have contributed to the following: Drafting the work or reviewing it critically for important intellectual content, final approval of the version to be published and agreement to be accountable for all aspects of the work in ensuring that questions related to the accuracy or integrity of any part of the work are appropriately investigated and resolved.

## CONFLICT OF INTEREST STATEMENT

The authors declare no conflict of interest.

## ETHICS STATEMENT

The research programme on laboratory test utilization (all phases), and the qualitative study to understand patient participants' perspective on laboratory testing in Phase 1 were approved by the institutional review board Conjoint Health Research Ethics Board of the University of Calgary (CHREB 17‐1215 and 20‐0728). Since all members of the PAC are active and equal members of the research team and consent to the publication of this manuscript, no specific ethics approval was required for this manuscript.

## Supporting information

Supporting information.

## Data Availability

The data presented in this study is available from the Patient Advisory Council, upon reasonable request in accordance with institutional policies and procedures.
