# Supplementary material for: Operationalizing the principles of patient engagement through a Patient Advisory Council: Lessons and recommendations
Source: Health Expect. 2023 Nov 9;27(1):e13909. doi: 10.1111/hex.13909 (PMC10726262; doi:10.1111/hex.13909)

Appendix Item 1:
Patient Partner Appreciation Guidelines: Compensation in Research

Prepared by the AbSPORU Patient Engagement Team

Purpose

Patients hold a vital role in patient-oriented research teams. Depending on their interest, skills, and available time, patient research partners can be essential collaborators to research priority setting, grant funding applications, study design, data collection, data analysis, knowledge translation, sharing of results, and evaluation (https://cihr-irsc.gc.ca/e/51910.html). In this document, we suggest how patient partners can be recognized financially for the essential lived experience and other expertise they bring to POR projects.

Our approach to patient partner compensation encompasses principles of respect, value, fairness, equity, inclusivity, responsiveness, and choice. These principles are aligned with Canadian Institutes of Health Research (CIHR) Supporting Patient-Oriented Research (SPOR). "Adequate support and flexibility are provided to patient partners to ensure that they can contribute fully to discussions and decisions. This implies creating safe environments that promote honest interactions, cultural competence, training, and education. Support also implies financial compensation for their involvement" (https://cihr-irsc.gc.ca/e/48413.html).

Patient partners contribute their essential lived experience expertise as active and equal members of research teams. Therefore, **patient partner compensation differs from 'tokens of appreciation' often given to participants** who contribute data to research projects (https://ethics.gc.ca/eng/policy-politique_tcps2-eptc2_2018.html) **and from reimbursement of direct expenses of engaging in a project or activity**. Patient partner compensation requires unique considerations and planning.

Scope

This is a living document subject to review in collaboration with patient partners on a regular basis to reflect trends and changes in health research contexts in Alberta. Because of the unique geographical and health care ecologies of the individual SPOR jurisdictions, each SPOR Support unit has co-developed their own patient partner compensation guidelines. We encourage you to reach out to the units through their respective websites. Links to respective SPOR SUPPORT Unit websites can be found here: https://cihr-irsc.gc.ca/e/45859.html.

Table 1. Glossary of Terms

**Patient**	An overarching term inclusive of individuals with personal experience of a health issue, and informal caregivers, including family and friends. See https://cihr-irsc.gc.ca/e/48413.html<br>*"Who is a patient?"* (60-second video)<br>https://www.youtube.com/channel/UCrLgpRV0t4CeOp69XyzPffQ
**Patient-Oriented Research (POR)**	A continuum of research that engages patients as partners, focuses on patient-identified priorities, and seeks to improve patient outcomes. This research, conducted by multidisciplinary teams in partnership with relevant stakeholders, aims to provide patient-centred evidence to improve healthcare systems and practices (https://cihr-irsc.gc.ca/e/48413.html). Depending on the context, patient-oriented research can also bring the collective voice of specific affected communities. See https://cihr-irsc.gc.ca/e/51910.html).
**Patient Engagement**	In Patient-Oriented Research, patient engagement describes patients engaged as active and equal research team members, beyond the role of participant. Patient partners collaborate early, often and in meaningful ways at any or all stages of the research process including priority setting, project design, grant applications, data collection, data analysis, knowledge translation, and evaluation. See https://cihr-irsc.gc.ca/e/48413.html
**Patient Partner**	Patients who collaborate as active and equal members of Patient-Oriented Research teams, in any or all the roles identified in the CIHR SPOR Health Research Cycle - see Part II in this document here: https://cihr-irsc.gc.ca/e/51910.html As members of health research teams, advising, providing feedback, and working together on research projects and activities through a lived-experience lens, this role does not require Research Ethics Board (REB) approval.
**Patient Participant**	A patient involved in a research study whose data, or responses to interventions, stimuli, or questions by a researcher, are relevant to answering the research question (https://cihr-irsc.gc.ca/e/51910.html) See also Glossary, Tri-Council Policy Statement: Ethical Conduct of Research Involving Humans, 2nd Edition (TCPS 2)<br>https://ethics.gc.ca/eng/policy-politique_tcps2-eptc2_2018.html
**Compensation (Patient Partner)**	Payment for the time, skills, lived experience and expertise patient partners bring to a research project or research related activity. Compensation is offered as recognition of the essential contribution patient partners bring to the research activity. There is no obligation to accept compensation. Compensation should not be understood as the market value of patient partner collaboration.

**Appreciation (Patient Participant)**	A token of appreciation, usually in the form of a pre-paid gift card or honorarium, given to a patient participant in a study or trial.
**Reimbursement**	Covering of the direct costs and expenses associated with engaging in research and research-related activities either as a patient partner or a patient participant. These include travel, parking, meals, care giving, digital access and other expenses and should be covered up front whenever possible.

Compensation Considerations

Compensation may not always be possible due to budget constraints. This can also be the case on grant applications and when funding has not been secured.

One size does not fit all, and each patient partner is a unique individual with personal preferences and circumstances. Types, amounts and methods of payments should be nimble, responsive, and flexible where possible.

Compensation is offered. There is no obligation to accept. It is essential to have a conversation about compensation at the beginning of working together in patient-oriented research. More about this compensation conversation can be found in the References section below.

Compensation offered to patient partners may have tax implications. Under the *Income Tax Act*, honoraria, gifts, or near-cash gifts are considered taxable income for which a T4A will be issued. Patient partners are encouraged to contact their financial advisor, accountant, or Canadian Revenue Agency. Discussions about tax implications arising from compensation are out of the scope of AbSPORU and Alberta Innovates.

These guidelines may have to be adapted to cover expenses for unique populations and communities including Indigenous Elders and Knowledge Keepers, i.e., flat fee rates or gifts. Consult with communities and institutions to determine appropriate and respectful approaches to recognition for populations with unique considerations.

Budget adequately for patient partner compensation in your grant application. Include compensation costs associated with dissemination including co-authorship and co-presentation of research findings at conferences, workshops, or forums.

Some institutions have maximum amounts for compensation. Patient partners paid more than this amount would be on contract or salaried, which is beyond the scope of these guidelines. Researchers are encouraged to check the policy and procedures of their organizations. Please reach out for our University of Calgary and University of Alberta Patient Partner Compensation guidance documents.

Conflicts of interest may arise in research when activities or situations place an individual in a real, potential, or perceived conflict between the duties or responsibilities related to the research, and personal, institutional, or other interests. It is important to identify, disclose, and manage real, potential, or perceived conflicts of interest. Conflict of interest policies are described in the relevant policies of all universities and Alberta Innovates.

Reimbursement Considerations

Patient partner compensation should not be confused with reimbursement of the direct expenses of contributing to a research project. Reimbursement of expenses associated with engaging in a research project or activity is necessary and should be a separate conversation from compensation. Direct expenses can include travel, parking, caregiving, digital access, training, and other similar costs.

These costs should be paid upfront by the research team as much as possible, rather than burdening the patient partner with the outlay of funds or added work of submitting an expense claim.

Each institution will have its own processes for pre-paying and reimbursing expenses. Be sure to check prior to engaging and having your patient partners incur expenses they can't be reimbursed for in a timely manner.

Table 2. Methods of Payment

Payment	CIHR defines payment as "the act of awarding something to someone in exchange for a service". Methods of payment can include salary, stipends, honoraria, in-kind exchanges and gift cards or gifts.<br>See our companion document for guidance on UCalgary payment processes
Fixed service	Related to a specific service (patient partners who invoice for services provided on a fixed rate)
In-kind compensation	Alternative forms of recognition including conference fees, co-authorship, training, invitation to special events, etc.
Honorarium	A one-time payment for cover costs for volunteer or guest speakers for which fees are not traditionally required
Pre-paid gift cards	Prepaid gift cards may still be considered income
For more information about payment methods and processes please see our University of Calgary and University of Alberta patient partner payment process guides here: https://absporu.ca/	

Table 3. Rates of Payment

Example of Engagement Activity		Suggested Compensation Rate
1	A one-time request to collaborate*	Hourly @ $25/hour<br>Half-day (+/- 4 hours) = $100<br>Full-day (+/- 8 hours) = $200
2	Member of working group, advisory council, or committee*	<4 times per year between $100-$200/year<br>>4 times per year between $200-$400/year
3	Partner on research project or activity<br>See Part II of this document<br>https://cihr-irsc.gc.ca/e/51910.html	Refer to #2 for standing collaborations<br>Refer to #1 for one-time or active collaboration
*It is essential that compensation is offered for pre-meeting activities such as reviewing agendas, documents and for time providing feedback through email communication.		

Please check https://absporu.ca/ for additional resources to support your patient partner compensation budgeting and processes.

Please reach out if you have questions or if you would like to learn more about our other supporting documents, resources and supports: **peplatformab@gmail.com**

References

Alberta SPOR SUPPORT Unit (AbSPORU) Website: https://absporu.ca/

Canadian Institutes of Health Research (CIHR), Considerations when paying patient partners in research https://cihr-irsc.gc.ca/e/51466.html

Canadian Institutes of Health Research (CIHR), Ethics Guidance for developing partnerships with patients and researchers https://cihr-irsc.gc.ca/e/51910.html

Canadian Institutes of Health Research (CIHR), Strategy for Patient-Oriented Research https://cihr-irsc.gc.ca/e/41204.html

Canadian Institutes of Health Research (CIHR), Strategy for Patient-Oriented Research – Patient Engagement Framework https://cihr-irsc.gc.ca/e/48413.html

Richards et al. (2022) Identifying potential barriers and solutions to patient partner (payment) in research

Richards et al. (2020) Patients as Partners in Research: How to talk about compensation with Patient Partners

Richards et al. (2018) Patient partner compensation in research and health care: the patient perspective on why and how

Tri-Council Policy Statement: Ethical Conduct for Research Involving Humans - TCPS2 (2018) https://ethics.gc.ca/eng/policy-politique_tcps2-eptc2_2018.html

AbSPORU
Alberta SPOR SUPPORT Unit

ALBERTANS 4 HEALTH RESEARCH

Appendix Item 2:
Compensation Submission Form, Patient Partners in Health Research

Prepared by the AbSPORU Patient Engagement Team

Patient partners are active and equal members of health research teams. Their collaboration is essential and valued, and as such compensation is offered for time and contribution. Conversations about compensation are important to have at the beginning of every research project, and need to include and address compensation amounts, roles and expectations, and forms of payment. Patient partners can always decline offers of compensation.
Compensation is distinct from reimbursement of direct expenses associated with engaging in research projects such as travel and parking.
More information about patient partner compensation can be found here:

Alberta SPOR Support Unit, Patient Partner Appreciation: Compensation in Research
CIHR, Considerations when paying patient partners in research

*Please complete this form and submit to the research team member who has been identified as the patient partner compensation liaison for the research project.

Patient Research Partner and Project Information

Patient Partner First and Last Name	
Project Title	
Principal Investigator	
Period of Engagement – months/year	

Activity Log

Activity Name -meeting, feedback, …	Description -roles, work done, etc.	Date of Activity	# of hours

Total Hours:			

Form of payment

___ I **do not** wish to accept payment for my time and contribution to this project.

___ I **do** wish to accept payment for my time and contribution to this project.

___ I wish to receive compensation in the form of a ___________________ gift card.

___ I wish this gift card to be online ___ or mailed via Canada Post ___.

Special Considerations

If you have made alternative arrangements than a gift card, for example, institutional human resources processes, in-kind tuition fees, or other arrangements, please detail below:

By signing below, you are acknowledging that you have completed the work outlined above and agree to be receiving recognition for this in the preferred, and agreed to, form of payment as outlined above.

Privacy and Confidentiality

Information provided will be strictly used for payment process purposes only and will be stored according to strictest privacy and confidentiality processes.

First and Last Name:	
Mailing Address:<br>-street, city, province, postal code	
Phone:	Email:
Signature:	Date:
Notes:	

Appendix Item 3:
Terms of Reference, RePORT PAC

Terms of Reference

Re-Purposing the **O**rdering of **R**outine laboratory **T**ests (RePORT) **P**atient **A**dvisory **C**ouncil (PAC)

**See Glossary of Terms for definition.*

Background of the RePORT study

The RePORT* study is a patient-oriented research* project which aims to co-design a stakeholder*-informed, healthcare provider* and patient partner* intervention bundle* to help address the overuse of laboratory tests in hospitals. This bundle will then be implemented, and its impact on the healthcare system, patients*, and healthcare providers will be evaluated. A patient engagement* strategy will be co-developed by a Patient Advisory Council (PAC) whose terms of reference can be found below.

PAC Purpose

The aim of the RePORT PAC is to help bring a diverse range of insights, perspectives, and reflections together to collaboratively gain a broader understanding of patient experiences of routine laboratory blood tests in a hospital setting.
The RePORT PAC members will work together to co-design in a range of patient engagement and research activities, for example:

- Providing feedback and insights to help guide the overall study
- Reviewing and editing documents
- Engaging in learning exercises
- Co-designing the interview guide
- Participant recruitment
- Assisting with interpreting data
- Assisting with Knowledge Translation*

In addition to the above activities, the PAC members may choose to be involved in other research-related work, for example:

- Taking part in training opportunities (Thematic analysis, NVIVO, conducting semi-structured interviews etc.)
- Data collection (eg. semi-structured interviews, focus groups, etc.)

- Data analysis
- Knowledge translation and dissemination* (e.g report writing, co-authoring, co-presenting on project findings and processes)

Membership

With the goal of equity, diversity, and inclusion in mind, the PAC will consist of:

- 8-10 patient partners
- Healthcare providers
- Research team members*
- Alberta Strategy for Patient Oriented Research SUPPORT Unit (AbSPORU) Patient Engagement (PE) Team* members

Co-chairs

The co-chairs are selected by the AbSPORU PE Team and the core research team. Co-chairs are three members from the RePORT Advisory Council and include two patient partners and one research team member. Should one of the co-chairs step down from their duties, the responsibilities shall be redistributed, decided collectively by the remaining members. The roles will evolve according to the needs of the council.

The co-chairs will be responsible for:

- Drafting and distributing the agenda for meetings
- Facilitating the meetings and discussions
- Ensuring Notes and Action Items are recorded
- Creating a safe space and allowing everyone to speak and be heard
- Guiding individual council members to work toward a common goal
- Working together as a team to resolve any conflicts and misunderstandings
- Providing support to interested advisory council members to collaborate on research related work, including data collection, analysis, and dissemination of findings

AbSPORU PE Team members will be responsible for:

- Supporting the establishment of the PAC
- Supporting the co-chairs when needed
- Attending PAC meetings and providing supports to the team

- Working with individuals of the team to track hours and providing compensation
- Process note taking*

Meetings and Information Sharing

All meeting materials and other resources will be stored in Google Drive.

Necessary meeting materials will be emailed, or upon request, mailed as a paper copy. Meetings will be held online over Zoom, with a phone-in option available.

PAC co-chairs will be available to address any questions or concerns. For contact information, see the Membership List.

- The PAC will meet monthly, with additional meetings as required.
- An agenda with relevant documents for the meeting, including previous meeting's notes, will be distributed by a PAC co-chair at least one week prior to the monthly PAC meeting date.
- The research team co-chair will be responsible for scheduling meetings via Doodle Poll*, sending meeting invites and hosting the Zoom session.
- A recording of each meeting will be made by the Zoom host and housed in the Google Drive.
- Notes and Action Items will be compiled by a PAC co-chair and circulated by email to PAC members shortly after each meeting. These will also be housed in the RePORT Google Drive.
- If a PAC member is unable to attend a meeting, please notify a co-chair by email.
- Reviewing items pre-meeting will save time and make for better meetings.
- Council members are expected to review the meeting Notes and documents from any missed meetings. Council members are encouraged to watch the video recording of any missed meetings. This time should be documented in the member's timesheet (the timesheet can be found on the RePORT Google Drive.
- Majority vote is not required. The PAC will strive to work by consensus in drafting its advice.

Compensation for Time and Reimbursement of Expenses for PAC Patient Partners

- Compensation will be offered, as per AbSPORU Guidelines* at a rate of $25 per hour for:
    - Each meeting attended
    - Compensation for 30 minutes of member's time will be offered for reviewing agendas and attachments prior to meetings.
    - Any work outside of meetings, for example:
        - Any additional preparation and review of documents or other material
        - Any other research tasks or activities outside of meeting hours assigned by the research team.
- The AbSPORU PE Team, PAC co-chairs, and the PAC patient partners will be responsible for tracking hours and compensation processes
- PAC members will be reimbursed for expenses incurred when meeting in person. These expenses may include:
    - Transportation (gas, mileage, parking, bus fare, taxi, airfare, etc.)
    - Food
    - Accommodation
    - Other expenses as approved by the research team
    - Large expenses (airfare, hotel, etc.) to be paid in advance so that the PAC members are not paying out of pocket.

Confidentiality

- All personal information is to be kept confidential.
- Research information is not to be shared outside of the RePORT PAC.
- If you are unsure of whether certain information is confidential, contact a co-chair for clarification or consult the RePORT PAC.

Stepping Down

Notification must be given to a RePORT PAC co-chair (via e-mail or phone).

Review of the ToR

This is a living document. Changes are expected and welcomed as the RePORT project progresses. This document was last edited on January 10, 2022.

Version History

Version 1; September 2, 2021

Version 2; November 15, 2021

Glossary of Terms

Listed alphabetically.

AbSPORU Patient Engagement (PE) Team: The Alberta Strategy for Patient Oriented Research SUPPORT Unit Patient Engagement Team provides ongoing support to patient, family, and community research partners, researchers, and community organizations to collaborate on health research priorities and studies. Enhancing the collaboration and capacity of all health research team partners contributes to meaningfully advancing the science and practice of patient engagement. https://absporu.ca/patient-engagement/

Doodle Poll: An online meeting scheduling tool (doodle.com).

Healthcare provider: A physician or other licensed individual who delivers healthcare services. https://www.albertadoctors.org/emr_resources/GlossaryofTerms.pdf

Intervention Bundle for RePORT study: The intervention bundle for RePORT includes two arms:

(i) Healthcare provide engagement strategy: this includes the use of an educational module, clinical decision support tool, and audit and feedback reports on the utilization of laboratory testing

(ii) Patient engagement strategy: this will include a patient-created infographic to help engage patients with the study and its objectives

Knowledge Dissemination: is the interactive process of communicating knowledge to target audiences so that it may be used to lead to change. The challenge is to improve the accessibility of desired knowledge products by those they are intended to reach. This means ensuring physical availability of the product to as large a proportion

of the target audience as possible and making the product comprehensible to those who receive it. https://link.springer.com/chapter/10.1007/978-981-10-0983-9_97

Knowledge Translation: A dynamic and iterative process that includes synthesis, dissemination, exchange and ethically-sound application of knowledge to improve the health of Canadians, provide more effective health services and products and strengthen the health care system. (*Source: Canadian Institutes of Health Research)*

Patient: An overarching term inclusive of individuals with personal experience of a health issue and informal caregivers, including family and friends. (*Source: Canadian Institutes of Health Research)*

Patient engagement : CIHR Strategy for Patient-oriented Research, Patient Engagement Framework
https://cihr-irsc.gc.ca/e/48413.html

Patient-Oriented Research: A continuum of research that engages patients as partners, focuses on patient-identified priorities and improves patient outcomes. This research, conducted by multidisciplinary teams in partnership with relevant stakeholders, aims to apply the knowledge generated to improve healthcare systems and practices. (*Source: Canadian Institutes of Health Research)*

Patient Partner: Individuals representing a variety of experiences, perspectives, and backgrounds who have lived experience of various health conditions and have received routine laboratory testing while in hospital. As equal members of the team, they bring valuable insights and knowledge to the research.

Patient Recruitment: Recruitment of patients to the RePORT study includes identifying eligible patients, explaining the study to potential participants, and obtaining informed consent to participate in the program and provide information for research.

Process Note Taking: notes, comments and reflections on the project and PE processes in addition to the meeting notes; the purpose of these process notes will be to inform potential co-developed process documents and publication manuscripts

Research Team Member: Staff members of the RePORT project who conduct research. This includes research assistants, research associates, the principal investigator, and the program manager/research scientist.

RePORT: Re-Purposing the Ordering of 'Routine' laboratory tests is a study that addresses the problem of laboratory test overuse in hospitals and proposes to develop an intervention bundle comprising a stakeholder-informed healthcare provider engagement strategy, and a patient co-designed engagement strategy. The

study will implement this bundle and evaluate its impact on the healthcare system, patients, and healthcare providers.

Stakeholder: An individual, group or organization having a "stake" in an issue and its outcome https://cihr-irsc.gc.ca/e/documents/ce_framework_e.pdf

Terms of Reference: A terms of reference document outlines the ways in which a group of people agree to work together to accomplish common goals *(Source: Health Quality Ontario)*
Timeline: Project funding ends in February 2022, with a one-year extension. We will re-apply for more funds March 2022

Patient and Family Advisory Council Work Flow

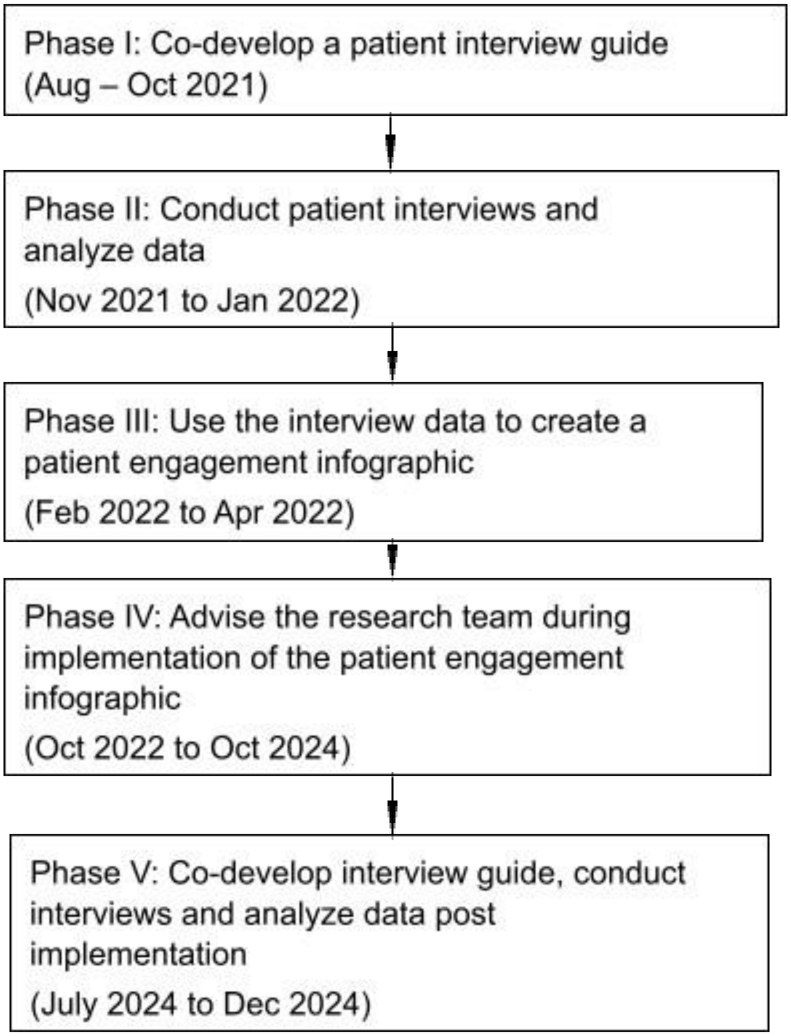

Supplement: Supplementary file 1 — Supporting information. [file HEX-27-e13909-s001.pdf]
